# Immune-Mediated Transverse Myelitis in a Filipino Patient With Prior Hematopoietic Stem Cell Transplantation: A Case Report

**DOI:** 10.7759/cureus.102119

**Published:** 2026-01-22

**Authors:** Jon Stewart H Dy, Ma. Cristina M Valdez, Francisco Lopez

**Affiliations:** 1 Neurosciences, St. Luke's Medical Center College of Medicine, William H. Quasha Memorial, Quezon City, PHL; 2 Neurosciences, St. Luke's Medical Center, Quezon City, PHL; 3 Bone Marrow and Transplant Service, St. Luke's Medical Center, Taguig City, PHL

**Keywords:** acute transverse myelitis (atm), autologous hematopoietic stem cell transplantation, immune-mediated myelitis, immunosuppression, spinal cord inflammation

## Abstract

Transverse myelitis is an inflammatory disorder of the spinal cord that can result in significant neurologic morbidity. Diagnostic evaluation is particularly challenging in immunocompromised patients because of a broad differential diagnosis that includes infectious, neoplastic, vascular, and treatment-related etiologies. We report the case of a 64-year-old Filipino male with a history of parotid carcinoma treated with surgery and radiotherapy, acute myeloid leukemia treated with hematopoietic stem cell transplantation, and chronic immunosuppression who presented with acute-onset progressive bilateral lower extremity weakness and sensory loss. Magnetic resonance imaging of the thoracic spine demonstrated focal intramedullary signal abnormalities at the first and second thoracic spinal cord levels. Extensive infectious, neoplastic, and inflammatory investigations were unrevealing. The patient was treated for a case of immune-mediated transverse myelitis with high-dose intravenous corticosteroids, resulting in substantial neurologic improvement. He achieved full motor recovery with residual sensory deficits following rehabilitation. There was no recurrence of myelopathic symptoms during follow-up. The patient later died from pneumonia in the setting of relapse of acute myeloid leukemia, without evidence of recurrent transverse myelitis. This case underscores the importance of systematic diagnostic evaluation and early initiation of immunosuppressive therapy in suspected immune-mediated transverse myelitis, even in patients with complex oncologic and immunologic histories.

## Introduction

Transverse myelitis is an acute or subacute inflammatory disorder of the spinal cord characterized by motor, sensory, and autonomic dysfunction [1]. Prompt recognition is essential, as early initiation of immunosuppressive therapy can significantly influence neurologic recovery and long-term functional outcomes [2]. In immunocompromised patients, including those with prior malignancy, exposure to cytotoxic therapies, or hematopoietic stem cell transplantation (HSCT), the diagnostic evaluation is particularly challenging due to a broad differential diagnosis encompassing infectious, neoplastic, vascular, and treatment-related etiologies [3,4].

We report a case of immune-mediated transverse myelitis in a Filipino male with a history of parotid carcinoma, hematologic malignancy treated with HSCT, and chronic immunosuppression. This case highlights the importance of systematic diagnostic reasoning and timely immunosuppressive intervention in identifying and managing transverse myelitis in patients with complex oncologic and immunologic backgrounds.

## Case presentation

A 64-year-old Filipino male presented with acute-onset, progressive weakness of the lower extremities in 2021. His past medical history was significant for parotid carcinoma stage IVB, for which he underwent radical parotidectomy in 2015, followed by cisplatin-based chemotherapy and radiotherapy administered over two months. He subsequently developed treatment-related myelodysplastic syndrome that progressed to acute myeloid leukemia. After achieving remission with induction and consolidation chemotherapy, he experienced recurrent bone marrow failure with hypoplastic myelodysplastic features and underwent HSCT in 2018. Since transplantation, he had been maintained on chronic low-dose prednisone 5 mg once daily for immune suppression. Additional medical history included herpes simplex virus infection in 2021, for which he was on acyclovir prophylaxis, and well-controlled hypertension.

The patient initially developed sudden weakness of the left lower extremity associated with numbness involving the entire limb. Over the succeeding seven days, weakness progressed and involved the right lower extremity. He denied bowel or bladder dysfunction, back pain, fever, weight loss, or other systemic symptoms. There were no reported cognitive changes, cranial nerve symptoms, visual disturbances, or upper extremity complaints. Prior to this illness, the patient was functionally independent and ambulatory without assistance. There was no recent history of infection, vaccination, trauma, or exposure to new medications preceding symptom onset.

On neurologic examination, the patient was alert with a Glasgow Coma Scale score of 15/15 and was oriented to person, place, and time. Cranial nerve examination revealed no abnormalities. Motor examination showed normal bulk and tone in the upper extremities, with full strength bilaterally. In the lower extremities, strength was reduced, with Medical Research Council grade 4/5 in the right lower extremity and 4+/5 in the left lower extremity [5]. There were no involuntary movements. Sensory examination revealed a clearly defined sensory level, with the last normal sensory level at the fourth cervical dermatome. On the left side, there was an approximately 20% reduction in sensation over the fifth cervical to first thoracic dermatomes, a 50% reduction over the first thoracic to twelfth thoracic dermatomes, and a 90% reduction over the first lumbar to second sacral dermatomes. On the right side, there was an approximately 20% reduction in sensation over the fifth to twelfth thoracic dermatomes and a 50% reduction over the first lumbar to second sacral dermatomes. Coordination testing revealed no cerebellar signs. Deep tendon reflexes were normoreflexic in the right upper extremity, hyporeflexic in the left upper extremity, and hyperreflexic in both lower extremities. There was no ankle clonus. Plantar responses were extensor bilaterally. Overall, the neurologic findings were consistent with an incomplete thoracic spinal cord syndrome, in keeping with an acute myelopathic process.

Given the patient’s acute presentation and complex medical history, the differential diagnosis for acute transverse myelopathy was broad and included infectious myelitis, neoplastic infiltration or relapse, radiation-induced myelopathy, vascular myelopathy, and immune-mediated transverse myelitis.

**Figure 1:**
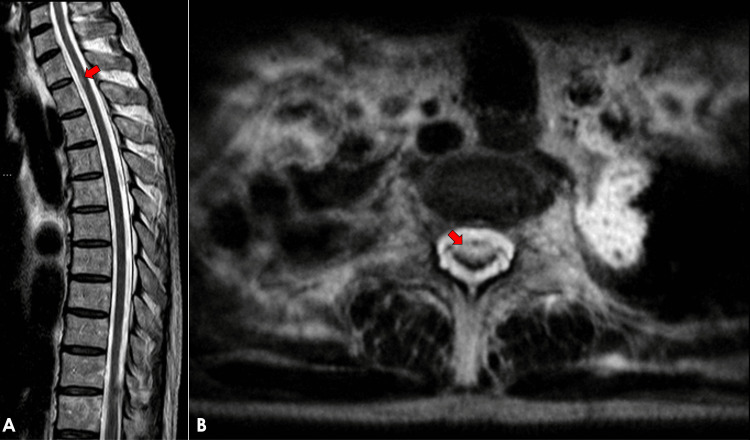
Magnetic resonance imaging of the thoracic spine demonstrating intramedullary spinal cord abnormalities. (A) Sagittal T2-weighted image showing a focal intramedullary hyperintense lesion at the level of the first and second thoracic spinal cord segments (arrow). (B) Axial T2-weighted image at the corresponding level demonstrating the intramedullary hyperintense signal within the spinal cord (arrow).

Magnetic resonance imaging (MRI) of the brain revealed no acute abnormalities, including absence of infarction, demyelinating lesions, or contrast-enhancing lesions. MRI of the thoracic spine demonstrated intramedullary hyperintense signal abnormalities on T2-weighted sequences at the first and second thoracic spinal cord levels, without associated cord expansion or contrast enhancement, suggestive of an inflammatory spinal cord process (Figure 1).

Initial laboratory evaluation did not reveal evidence of systemic infection, metabolic derangement, or inflammatory disorder. Complete blood count, serum electrolytes, and renal and hepatic function tests were within normal limits. There was no laboratory evidence suggestive of acute infection, metabolic myelopathy, or hematologic relapse at presentation.

Lumbar puncture revealed normal opening and closing pressures. Cerebrospinal fluid (CSF) analysis showed normal cell count, protein, and glucose levels. Oligoclonal bands were absent, and the immunoglobulin G index was within normal limits. Cytology and cell block analysis demonstrated no malignant cells. Comprehensive infectious studies, including bacterial, viral, fungal, and mycobacterial testing, were negative. Overall, CSF findings showed no evidence of infection, malignancy, or alternative inflammatory pathology.

Infectious and neoplastic etiologies were considered unlikely based on the absence of systemic symptoms, negative CSF studies, and lack of imaging features suggestive of infiltrative disease. Radiation-induced myelopathy was considered but deemed less likely given the acute onset, imaging characteristics, and subsequent response to corticosteroid therapy. Vascular causes such as spinal cord infarction were also unlikely due to the subacute progression of symptoms and lack of diffusion-restricted lesions. Taken together, the clinical presentation, the absence of systemic laboratory abnormalities, radiologic findings, CSF profile, and systematic exclusion of alternative etiologies supported a diagnosis of immune-mediated transverse myelitis.

The patient was treated with high-dose intravenous methylprednisolone, 1 gram per day, for five days. Supportive care and early mobilization were initiated concurrently. Following completion of corticosteroid therapy, the patient demonstrated improvement in lower extremity motor strength. Pertinent neurologic examination after pulse steroid therapy showed improved strength in both lower extremities. A sensory level persisted, with partial recovery of sensation. Reflex examination remained normoreflexic in the right upper extremity, hyporeflexic in the left upper extremity, and hyperreflexic in both lower extremities, with persistent bilateral extensor plantar responses. The patient was discharged with a structured physical rehabilitation program. Over the subsequent six months, he achieved full recovery of motor strength in all extremities. Residual left-sided sensory deficits persisted but gradually improved with rehabilitation, with no recurrence of myelopathic symptoms during follow-up.

Approximately 2.5 years after the neurologic event, the patient died from pneumonia in the setting of relapse of acute myeloid leukemia. There was no clinical evidence of recurrence or progression of transverse myelitis prior to death.

## Discussion

Transverse myelitis is a heterogeneous inflammatory disorder of the spinal cord with diverse etiologies, including autoimmune, infectious, paraneoplastic, vascular, and treatment-related causes [4,6-10]. Establishing the diagnosis is particularly challenging in immunocompromised patients, in whom atypical presentations and overlapping risk factors substantially broaden the differential diagnosis [3]. This case underscores the importance of systematic diagnostic evaluation and careful clinical reasoning in identifying immune-mediated transverse myelitis in patients with complex oncologic and hematologic histories.

Infectious myelitis was a major diagnostic consideration given the patient’s immunocompromised state; however, the absence of systemic symptoms, normal CSF parameters, and negative infectious studies argued against this etiology [7]. Neoplastic causes, including leukemic relapse or paraneoplastic spinal cord involvement, were also considered but were not supported by CSF cytology, cell block analysis, neuroimaging findings, or the subsequent clinical course [8]. Radiation-induced myelopathy was likewise considered in view of the patient’s prior radiotherapy [9]. Delayed radiation myelopathy typically presents months to years after exposure and is characterized by progressive neurologic decline with limited response to immunosuppressive therapy [9]. In contrast, the acute onset of symptoms, focal inflammatory-appearing spinal cord lesions on MRI, and favorable response to corticosteroid treatment in this case argued against radiation-related injury [9]. Vascular etiologies, including spinal cord infarction, were considered less likely given the subacute symptom progression, absence of acute pain, and lack of diffusion restriction on imaging [10].

The patient’s history of HSCT, exposure to cytotoxic chemotherapy and radiotherapy, and prolonged immunosuppression necessitated careful consideration of rare post-transplant neurologic complications, including immune-mediated transverse myelitis [3]. Transverse myelitis following HSCT is a rare but increasingly recognized complication, reported predominantly in isolated case reports, most commonly after allogeneic transplantation [3]. Proposed mechanisms include immune dysregulation during immune reconstitution, donor T-cell-mediated neuroinflammation, cytokine-driven spinal cord injury, and central nervous system manifestations of graft-versus-host disease (GVHD) [11]. Although GVHD classically affects the skin, liver, and gastrointestinal tract, neurologic involvement has been described and may affect both the peripheral and central nervous systems [11]. In this case, the temporal relationship to prior transplantation, exclusion of alternative etiologies, and favorable response to corticosteroid therapy were most consistent with an immune-mediated inflammatory process affecting the spinal cord [3,6,11]. While CSF studies were unremarkable, normal CSF parameters do not exclude immune-mediated myelitis and have been reported in a subset of cases [11]. The absence of concomitant brain MRI abnormalities further supported a spinal cord-predominant inflammatory process rather than a disseminated demyelinating disorder [6].

High-dose corticosteroids remain the first-line treatment for immune-mediated transverse myelitis and were associated with meaningful neurologic recovery in this patient [12]. Consistent with prior studies, motor improvement preceded sensory recovery, with residual sensory deficits persisting despite restoration of strength [13]. This pattern highlights the importance of early immunosuppressive therapy and sustained rehabilitation in optimizing functional outcomes [12,13].

Although the patient ultimately died from pneumonia related to relapse of acute myeloid leukemia, there was no evidence of recurrence or progression of the myelopathic process during follow-up. This distinction is clinically important, demonstrating that neurologic recovery from transverse myelitis can be achieved even in patients with severe systemic illness and significant comorbidities [3].

Overall, this case reinforces the need for heightened clinical vigilance for immune-mediated neurologic complications following HSCT. Early recognition, thorough exclusion of secondary causes, and prompt initiation of immunosuppressive therapy remain critical to improving neurologic outcomes in this high-risk population [3,6,12,13].

## Conclusions

Immune-mediated transverse myelitis is a rare but important neurologic complication following HSCT and should be considered in immunocompromised patients presenting with acute spinal cord syndromes after exclusion of infectious, neoplastic, vascular, and treatment-related causes. Awareness of this potential post-transplant manifestation is essential for timely diagnosis. Prompt recognition and early initiation of immunosuppressive therapy may result in meaningful neurologic recovery, even in patients with complex comorbid conditions and advanced systemic disease.
